# Paranormal beliefs and individual differences: story seeking without reasoned review

**DOI:** 10.1016/j.heliyon.2020.e04259

**Published:** 2020-06-30

**Authors:** Tilmann Betsch, Leonie Aßmann, Andreas Glöckner

**Affiliations:** a Department of Psychology, University of Erfurt, Germany; b Department of Psychology, University of Cologne, Germany; c Max Planck Institute for Research on Collective Goods, Bonn, Germany

**Keywords:** Psychology, Paranormal beliefs, Esoterism, Personality, Individual Differences

## Abstract

In a sample of 599 participants (60% female, 18–81 years), we tested the hypotheses that cognitive ability and the big-six personality traits suffice to explain the individual-difference component of paranormal beliefs (belief in magic, astrology, esoterism, supernatural beings, and spirituality). Additionally, we measured 14 other potential predictors that were found to correlate with paranormal beliefs in prior research (e.g., ontological confusion). Although cognitive ability and the big-six explained 10% of the variance in individual belief, ontological confusion and causality understanding also were significant predictors in regression analyses. The resulting model, explaining 19 % of variance, consists of ontological confusion, cognitive ability (negative correlation), openness to new experiences, emotionality, conscientiousness (neg. corr.) and causality understanding (neg. corr.). We discuss the findings with reference to two hypothetical factors that drive individuals' acceptance of paranormal beliefs, inclination for story-telling, and tendency to evaluate belief content in terms of reason and conscientious evaluation.

## 1 Introduction

The homo sapiens is capable of telling fictions, i.e. stories about entities or phenomena that cannot be seen, heard, touched, or smelled (Harari, 2015). If Carla destroys Tom's sandcastle and Tom's mother subsequently complains about “that *aggressive* girl at the playground”, this would be an example of a fictitious story. According to attribution research (Uleman and Bargh, 1989), this judgment reflects a so-called spontaneous trait inference (aggressiveness) based on observation of a behavior (destroying a sand castle). Individuals tend to “explain” behavior based on internal causes such as the other person's personality. Personality, however, cannot be directly observed. It is a hypothetical construct. Nevertheless, Tom's mother may be confident that her belief about Carla's personality reflects the truth. Even if we doubted the idea that Carla truly is aggressive, we would not label the mother's belief as paranormal, because the notion that traits can predict behavior converges with theory and empirical evidence in academic psychology. However, although we generally accept stories referring to stable individual differences, we may consider a single observation an invalid measure of Carla's personality. Still, in principal it would be possible to subject Carla to a standardized personality test, which represents a widely accepted means with which to measure the distal entity of a trait.

Assume, however, that Tom's mother believed that an evil magician casted a spell on Carla to make her aggressive. Although this, too, represents a fictitious story, most individuals would likely find it bizarre – and one may describe it as a story reflecting paranormal beliefs. We define paranormal beliefs as *non-religious* fictitious stories about entities, mechanisms, and practices that contradict established scientific knowledge (cf., van Prooijen et al., 2017; see Lindeman and Svedholm, 2012 for a discussion of the diversity of definitions). We use a scale of paranormal beliefs that considers beliefs in magic, astrology, esoterism, supernatural beings, and spirituality (see method section). In a large, representative German survey (GESIS - Leibniz Institute for the Social Sciences, 2013), 25–50% of the population believed in the validity of paranormal phenomena and pseudo-scientific healing methods to at least some extent. The services and products in the paranormal domain have an estimated annual turnover of more than 15 billion Euros in Germany alone (Klaus, 2017; see also Potten and Memminger, 2017).

What types of people endorse paranormal beliefs? In the US, low-income, highly religious females appear to be most likely to hold strong paranormal beliefs (Chapman University, 2017, but see Krull and McKibben, 2006; Reiner and Wilson, 2015 for contradicting results). Psychological research identified a plethora of other predictor variables. Some of the findings indicate that cognitive ability and knowledge are important factors. Paranormal believers were found to be low performers in the educational system (Musch and Ehrenberg, 2002) and especially susceptible to commit reasoning errors (Rogers et al., 2009) as well as having a tendency to think in a superfluous, non-analytical manner (Gray and Gallo, 2016).

Table A in the Appendix contains examples of studies. In addition to the diversity of predictors, the most striking observation is that researchers typically focus on a *small selection* of factors. Unfortunately, the relative predictive power of each individual factor remains unknown if it is not assessed in comparison to other potential factors. Interestingly, many studies refrain from comparing their focal predictors to the most commonly used personality model in psychology, the Big Five (e.g., Goldberg, 1992). Those who do consider the standard personality model exclude potential competitors (e.g., Henningsgaard and Arnau, 2008) or include only a small subset of other variables (e.g., Schnell, 2012). For decades, conscientiousness, openness to experience, agreeableness, extraversion, and neuroticism have been widely used to describe personality. However, more recently, a six-factor solution has been suggested. Rather than measuring neuroticism, the Hexaco inventory includes the dimensions honesty-humility and emotionality (Ashton and Lee, 2007). There is compelling evidence from a growing body of data collected from all over the world that the six factor solution accounts for most of the variance in personality (cf., Lee and Ashton, 2018a, Lee and Ashton, 2018b).

In the current study, we examine rather stable individual differences as predictors of paranormal beliefs^1^. We will compare numerous individual variables with the HEXACO personality traits in terms of their predicative power regarding paranormal beliefs. We aim to replicate bivariate correlations documented in the literature to date. However, we expect only few predictors to remain significant in a competitive analysis that also contains the Big Six and standard measures of cognitive ability such as IQ. Specifically, we hypothesized that:

(H1) the Big Six suffice to account for the personality component in paranormal beliefs.

An additional cluster of individual differences refers to ability. As already noted, prior research indicates that cognitive ability is negatively correlated with the extent to which people believe in paranormal issues. Thus, we hypothesize that:

(H2) cognitive ability is a significant predictor of paranormal beliefs in addition to the Big Six.

## 2 Method^2^

### 2.1 Participants

Participants were recruited from a large participant pool^3^ established by the University of Hagen, Germany. Among other variables, personality measures (HEXACO), cognitive ability (IQ), and level of education were already assessed in a basic survey prior to the current study. Based on an a priori power analysis (G-Power, Faul, Erdfelder, Buchner & Lang, 2009) we aimed to sample 600 participants in order to be able to detect small effects (Cohen's *f* ^2^ = .02) with 95 % statistical power (linear regression, single coefficient, alpha = .05, one-sided test). In a predetermined period of data collection, 599 individuals participated in the study. None were excluded from subsequent analyses. The sample contained 362 women (60 %) and 237 (40%) men – thus slightly overrepresenting females (51% in the German population, Statistisches Bundesamt, 2017). Age ranged between 18 und 81 years with a mean age of 33.63 years (*SD* = 11.38), so that our sample was ten years younger than the population average (44.4 years in 2018; Statista, 2019). Cognitive ability in the sample (mean IQ = 100, *SD* 14.99) converged with the mean in the population (mean IQ = 99, Lynn and Meisenberg, 2010). Forty-two percent of participants reported holding an academic degree, so that education in the sample was higher than in the German population (21 % academics, Statistisches Bundesamt , 2017).

### 2.2 Materials

Researchers have employed a wide range of measures to assess belief in the paranormal. Although the Revised Paranormal Belief Scale (RPBS, Tobacyk, 2004) is the most widely used (Goulding and Parker, 2001), there is an ongoing debate regarding its validity and factorial solutions (e.g., Drinkwater et al., 2017). The content, scope, and prevalence of paranormal subjects change over time and differ between cultures, even among Western countries (Sen and Yesilyurt, 2014). The New Age component of the RPBS (Lange et al., 2000) contains three (out of eleven) items that address psychokinesis. This was a prominent issue back in the 1970/80s when Uri Geller bended spoons in TV shows. Today, however, other topics dominate the esoteric market, such as energy healing and energy work, which are rarely addressed in older scales.

In order to identify the paranormal issues that are topical for our German target population, we invested considerable effort in exploring the paranormal market. Specifically, we analyzed contents in social media, online shops for esoteric products, and print media in addition to interviewing visitors of esoteric/spiritual fairs across the country. We identified five subject areas corresponding to five items. Each item names the category and prompts the semantic space by a list of exemplars (see supplement). On six-point rating scales, participants are asked how much they believe in esoterism (e.g. reiki, energy healing), spirituality (e.g. channelling, past life regression), supernatural beings (e.g. ghosts, light beings), astrology (e.g. horoscope, tarot cards), and magic (e.g. witchcraft, voodoo). The scale satisfies the core aspect of the construct as all exemplars violate scientific knowledge. Moreover, with our inductive approach, the semantic space of the scale is likely to be representative for what German speaking people are likely to encounter when they gather information about the paranormal on the internet, in media and at fairs.

Ideally, a competitive test should include all variables that have been found to correlate with paranormal beliefs in prior research. Practically, this is not possible. In addition to the sheer number of variables, authors employed different scales to measure the same construct (e.g. numeracy was measured as base-rate neglect, deviations from expected value, Dagnall et al., 2007; Dagnall et al., 2014), conjunction fallacy (Brotherton and French, 2014; Dagnall et al., 2007; Prike et al., 2017), and perception of randomness (Dagnall et al., 2007; Dagnall et al., 2016). To approach a finite set of variables, 26 scales were tested in correlative online-studies with almost 2,000 participants overall (Aßmann, 2017; Gildehaus, 2017; Hansmann, 2017; Klodt, 2017; Wünsche, 2017). Those studies also pre-tested the reliability of our paranormal belief scale, which resulted in an acceptable reliability ranging between .81 to .92 (Cronbach's alpha).

Note that each of these studies focused on a subset of potential predictors for paranormal beliefs. We included only those variables in the current study which significantly correlated with paranormal belief in the pre-studies. Those were the Big Six (Honesty/Humility, Emotionality, Extraversion, Agreeableness, Conscientiousness, Openness to New Experiences and Altruism), Cognitive Ability (IQ), Education, Ontological Confusion^4^, Numeracy, Causality Understanding, Ambiguity Tolerance, Need for Cognitive Closure, Epistemological Prudence, Need for Cognition, Life Satisfaction, Illusory Pattern Perception, Death Anxiety, Cognitive Style, Age and Sex.

TableI (supplement) provides not only the resulting list of potential predictor variables used in this study but also example items.

### 2.3 Procedure

The questionnaire comprised 121 items in a fixed order and was conducted online using UniPark EFS Surveys. Participants voluntarily agreed to participate in a study on attitudes and beliefs (informed consent was obtained in prior registration of the pool). They received a flat fee of € 5 in compensation for their participation. This research did not receive any specific grant from funding agencies in the public, commercial, or not-for-profit sectors and was approved by the University of Erfurt Ethic Board.

## 3 Results

Reliability of the majority of scales ranged between .76 and .92 (Cronbach's alpha). Lower alphas were obtained for epistemological prudence (.59) and death anxiety (.66). Overall, we replicated most of the correlations reported in the literature (see Tables B-D, Appendix) with the exception of life satisfaction (Gray and Gallo, 2016) and some personality traits (e.g., extraversion and agreeableness; Henningsgaard and Arnau, 2008; Schnell, 2012). These findings indicate that the majority of the variables assessed in our study represent promising candidates for predictors of paranormal beliefs. Descriptive statistics of all relevant variables can be found in Table E in the Appendix.

We hypothesized that the Big Six (H1) together with cognitive ability (H2) sufficiently account for individual differences in paranormal beliefs. To test these hypotheses, we first performed a linear regression analysis with paranormal beliefs as the criterion and the Big Six and IQ as predictors to assess how much variance is explained by those factors alone. Because this is a confirmatory analysis, we entered all predictors simultaneously into the regression model. Table 1 displays the results. The model with the seven predictors explained approximately 10 % of the variance in paranormal beliefs. As expected, cognitive ability (IQ) also correlates with the criterion. Emotionality and openness to experience correlate positively, whereas conscientiousness and IQ correlate negatively with the tendency to hold paranormal beliefs.

**Table 1:** Results of regression analysis with personality traits (Big Six) and cognitive ability (IQ) as predictors and paranormal belief as criterion.

	B	SE	Beta	T	p
(constant)	2.024	.499		4.060	.000
Honesty-humility	-.034	.059	-.025	-.587	.558
Emotionality	.237	.069	**.141**	3.441	**.001**
Extraversion	.094	.063	.063	1.489	.137
Agreeableness	.089	.072	.053	1.242	.215
Conscientiousness	-.284	.071	**-.162**	-3.981	**<.001**
Openness	.182	.068	**.111**	2.693	**.007**
IQ	-1.345	.260	**-.207**	-5.170	**<.001**

Note*: F*(7, 591) = 9.738; *p* < .01; *R* = .322, *R*^*2*^ = .103, *SE* = .946. Bold value indicates significant at the .05 level (2-tailed).

In a second step to test our hypotheses, we included all predictors together in a linear regression. As displayed in Table 2, IQ and emotionality no longer significantly predict the criterion. In addition, four other factors become significant with the strongest effects for sex and ontological confusion. Clearly, the results refute our hypothesis. That is, the Big Six and IQ do not suffice to account for individual difference in paranormal beliefs, because the saturated model explains more than twice the variance (24.6 %).

**Table 2:** Results of a regression analysis with all predictors and paranormal belief as criterion.

	B	SE	Beta	T	p
(constant)	1.333	.879		1.517	.130
Honesty-Humility	-.096	.064	-.069	-1.494	.136
Emotionality	.086	.084	.051	1.019	.308
Extraversion	-.010	.066	-.007	-.148	.882
Agreeableness	.127	.076	.075	1.678	.094
Conscientiousness	-.241	.071	**-.137**	-3.387	**.001**
Openness	.228	.068	**.138**	3.340	**.001**
IQ	-.351	.313	-.054	-1.122	.262
Altruism	.043	.085	.029	.503	.615
Education	-.004	.028	-.005	-.129	.897
Ontological Confusion	.434	.068	.**257**	6.388	**<.001**
Numeracy	-.028	.022	-.056	-1.276	.203
Causality Understanding	-.077	.035	**-.086**	-2.217	**.027**
Ambiguity Tolerance	.063	.071	.047	.885	.376
Need for Cognitive Closure	-.071	.082	-.050	-.872	.384
Epistemological Prudence	-.059	.064	-.040	-.911	.363
Need for Cognition	-.007	.053	-.006	-.122	.903
Life Satisfaction	.005	.017	.013	.316	.752
Illusory Pattern Perception	.031	.029	.041	1.065	.287
Death Anxiety	.035	.015	**.100**	2.362	**.018**
Cognitive Reflection Test	-.039	.039	-.045	-.996	.320
Age	-.002	.004	-.021	-.526	.599
Sex	.387	.086	.**191**	4.515	**<.001**

Note*: F*(22, 598) = 8.530; *p* < .01; *R* = .496, *R*^*2*^ = .246, *SE* = .879. Bold value indicates significant at the .05 level (2-tailed).

It is surprising that two predictors (emotionality, IQ) are not significant in the second regression, especially because IQ had the highest beta in the first regression. Sex is a strong predictor of paranormal beliefs. IQ and sex were strongly correlated (*r* = -.245, *p* two-tailed < .001), indicating that these two variables are confounded in our sample such that the female subsample is significantly lower in IQ than the male subsample (*F* (1, 598) = 38.245; *p* < .01)^5^. To cope with collinearity, we decided to run an additional regression without sex as a predictor. Since this is an explorative analysis, we used a stepwise regression in order to assess the best grouping of predictor variables that account for the most variance in the criterion variable.

Table 3 shows the results. The saturated model comprises six predictors accounting for 19 % of the variance in paranormal beliefs. It contains all predictors that significantly explained variance in the first regression – IQ as well as the traits openness, emotionality and conscientiousness. Notably, however, two additional variables were significant in the model – ontological confusion and causality understanding. Ontological confusion is now the strongest predictor, accounting for approximately 8 % of the variance.

**Table 3:** Model solution from a stepwise regression analysis including all predictors except sex and paranormal beliefs as criterion.

	B	SE	Beta	T	p
(constant)	2.024	.499		4.060	.000
Ontological Confusion	.494	.065	**.292**	7.631	**<.001**
IQ	-1.031	.250	**-.158**	-4.127	**<.001**
Openness to New Experience	.230	.062	**.140**	3.708	**<.001**
Emotionality	.215	.063	**.128**	3.422	**.001**
Conscientiousness	-.211	.066	**-.120**	-3.223	**.001**
Causality Understanding	-.081	.034	**-.089**	-2.364	**.018**

Note*: F*(6, 598) = 23.632; *p* < .01; *R* = .440, *R*^*2*^ = .193, *SE* = .897. Bold value indicates significant at the .05 level (2-tailed).

To check for the robustness of these findings, we randomly split our sample into an exploratory sample (n = 300) and a validation sample (n = 299). The stepwise regression analyses yielded widely similar but not identical results. In the exploratory sample, the saturated model accounted for 19.1 % of the variance (*F* (5, 294) = 13.842; *R* = .437; *SE* = .912) and contained five predictors: ontological confusion (*beta* = .287; *p* < .01), IQ (*beta* = -.157; *p* < .01), openness (*beta* = .140; *p* < .05), emotionality (*beta* = .138; *p* < .05), and causality understanding ((*beta* = -.107; *p* < .05). In the validation sample, the saturated model accounted for 18.7 % of the variance (*F* (5, 293) = 13.486; *R* = .433; *SE* = .900) and also contained five predictors: ontological confusion (*beta* = .271; *p* < .01), IQ (*beta* = -.177; *p* < .01), openness (*beta* = .172; *p* < .01), causality understanding ((*beta* = -.119; *p* < .05), and conscientiousness (*beta* = .107; *p* < .05). The two analyses together replicated the predictor solution from the stepwise regression of the total sample with a comparable amount of variance explained. However, two of the Big-Six factors, emotionality and conscientiousness, did not consistently enter the model. With this caveat, the model solution replicated quite well in the split-half approach. Most importantly, results further validate the refutation of our hypothesis stating that the Big Six and cognitive ability would suffice to explain the individual difference component of paranormal beliefs. Still, these variables do explain a substantial portion of the variance in the criterion.

For further interpretation of the multiple regression results, we conducted a dominance analysis (Budescu, 1993; Johnson, 2000). We used the SPSS-MIMR-cor program (Lorenzo-Seva et al., 2010) to determine Johnson's relative weights for the importance of predictors (see OSF repository for details). The results showed that ontological confusion's relative contribution to Multiple R was 47.8%. Cognitive ability accounted for 18 %, personality for altogether 25% (openness 6.2 %, emotionality 9.9%, conscientiousness 8.9%), and causality understanding for 9.1%.

## 4 Discussion

We show that personality traits and cognitive ability (IQ) explain a portion of individual differences in paranormal beliefs. Certainly, neglecting these variables would produce a biased picture of the evidence. The literature has examined a large number of variables that correlate with paranormal beliefs in the absence of Big Six and IQ. In a comparison of all variables, however, only six factors remained significant, including three personality traits (openness to experience, emotionality, conscientiousness) and IQ as a measure of general cognitive ability. Nevertheless, these factors do not sufficiently describe the differences between individuals believing in, say, magic, channeling, or reiki. As such, the results falsified our hypotheses that paranormal beliefs could be regressed to the Big Six and IQ without necessitating the inclusion of other individual differences in the model. An additional factor also appears to be of paramount importance – ontological confusion. With respect to this variable, we replicated large-scale research by Lindeman et al. (2015) who introduced this concept to the literature on belief in fictions (Lindeman and Saher, 2007; Lindeman and Aarnio, 2007). Moreover, causality understanding was added to the set of six variables that, altogether, accounted for almost 20% of the variance in paranormal beliefs.

What is characteristic of individuals who hold paranormal beliefs? According to our findings, individuals with paranormal beliefs tend to confuse ontological categories, have lower cognitive ability, be open to new experiences, be emotional, be reluctant to think in a thorough fashion (low conscientiousness), and neglect control groups (low causality understanding). In descending order, this list mirrors the inclusion of each factor into the model contingent on the amount of additional variance explained. Considering the factors in more detail, one may group them into two categories that concern the construction of reality in a story-like fashion and the avoidance of reasoned review.

### 4.1 Stories

Individuals with ontological confusion tend to view metaphors as literal facts. This “ontological” confusion results from applying one domain of reality (e.g., living beings) to another non-applicable domain (e.g., lifeless objects). In the beginning of attribution research, researchers found that individuals described movements of geometrical figures in terms of social actors and behavior (Heider and Simmel, 1944). Jerome Bruner (1957) nicely coined this as an instance of “going beyond the information given”. Encoding involves establishing a relationship between a perceptual input and the coding systems – i.e., our knowledge about the world, exemplars, categories, and schemata stored in long-term memory. As such, each output of an encoding episode is the result of an interaction between the stimulus and our knowledge. In the course of this process, we create meaning. Without meaning, we could not grasp what is happening in the environment around us.

In a series of studies on pseudo-profound bullshit, Pennycook and colleagues (Pennycook et al., 2015, p. 550–552) asked participants to rate how *profound* statements such as “attention and intention are the mechanism of manifestation” are. They defined profound as “of deep meaning; of great and broadly inclusive significance”. They found that people who believed in the paranormal also showed high bullshit receptivity scores; i.e., they were prone to view such communications as being profound. Moreover, the authors reported a strong negative correlation between bullshit receptivity and analytic thinking, whereas bullshit receptivity was strongly positively related to ontological confusion. Commenting on Pennycook et al.'s findings, Dalton (2016) questioned the validity of the construct. Bullshit receptivity and ontological confusion may not be “value- or culture-neutral”. They may “support a less restrained openness to experience but lead to ‘failure’ from a Western analytical perspective”.

Notably in our study, openness to experience significantly predicts paranormal beliefs. Paranormal believers report themselves on the HEXACO as having a good imagination, and being an artistic or creative type. Any creative cognition involves an expedition far beyond the given, as does the pursuit of a deeper understanding or meaning. It may conflict on occasions, however, with the application of rules that require selective consideration of information and the rigidity of applying a stopping rule. As such, people open to new ideas may be prone to transgressing points of truncation required by formal rules.

As another factor promoting paranormal beliefs, we found that emotionality is positively correlated with paranormal beliefs. Emotionality also ties into the interpretation that people may differ in terms of how they tend to conceive and approach their environment. An appealing narrative not only offers insight and understanding but also evokes affective reactions. Fischler (2017) compiled numerous examples of how new-age agents utilize emotion when marketing their products and services. In essence, they sell affectively compelling stories.

### 4.2 Review

The three factors ontological confusion, openness, and emotionality may potentially be considered facets of a tendency in individuals to prefer stories, i.e. vivid and affectively appealing conceptions of the world. This is not a deficit per se. Many of us enjoy such stories as presented in books or TV series. Nothing is wrong with that. However, believing that the story characters truly exist might be associated with some psychological problems. Hence, ensuring the accuracy of our beliefs does have some adaptive value. Accordingly, we must be able to distinguish between conceptions of the world that differ in their validity. This, in turn, requires ability and motivation to review claims in a reasoned fashion. And surely, ability and motivation for examination of propositions are also necessary in all domains of belief formation.^6^ Our results show that belief in the paranormal is negatively correlated with general thinking ability. Among other aspects of problem solving, our IQ-test primarily measures logical understanding and rule application (mathematical rules, rules for category inclusion/exclusion, etc.). Moreover, those people who tend to hold paranormal beliefs scored low on conscientiousness. A large number of items of Hexaco's conscientiousness subscale address accuracy motivation and the tendency to organize action and cognitive activities in a planned fashion (Lee and Ashton, 2018a). Another facet is causality understanding. The item describes an incidence of a seemingly causal relationship. Participants were asked whether the coincidence of two things suffices as proof of a causal relationship. Moreover, they are presented with methods for testing causality, which resemble an experimental design. Individuals believing in paranormal issues tend to deny the fact that a rigid test of causal relationships requires a control-group approach. So, believers in the paranormal appear to have lower cognitive ability, motivation, and formal skills to review conceptions in a reasoned fashion.

### 4.3 Limitations

We used a self-constructed scale for the assessment of paranormal beliefs. Although our scale is not yet formally validated, we could successfully replicate a bulk of bivariate correlations reported in the literature (see supplement). Notably, measures of paranormal beliefs also varied in prior studies. For example, Lindeman et al. (2015) reported a strong correlation between ontological confusion and the Revised Paranormal Belief Scale (RPBS, Tobacyk, 2004). Musch and Ehrenberg (2002) showed that cognitive ability and paranormal beliefs were negatively correlated. They constructed a scale that was partly based on Glicksohn (1990) scale. Henningsgaard and Arnau (2008) found a covariation between some Big Five factors such as consciousness with the Spirituality Meaning Scale (Mascaro et al., 2004). Consciousness also correlated with Tobacyk's RPBS in a study by Williams and Roberts (2016). Convergence between results from these and our study suggests that our scale was quite effective in measuring the construct of paranormal beliefs. We focused on individual differences, i.e. personality, and other rather stable dispositions such as cognitive ability.

Regression analyses revealed a model containing six factors explaining approximately 20 % of the variance in paranormal beliefs. On the one hand, this finding shows that individual differences are indeed important factors. On the other hand, they are not sufficient to understand the phenomenon. What factors drive the 80 % variance unexplained by individual differences?

Psychologist have examined the functional side of *religious* beliefs for more than one hundred years (e.g., James, 1902; Gebauer et al., 2012). The adaptive value of paranormal beliefs, however, has received comparatively little consideration. Although assuming that magical/delusional thinking is fostered by misattribution, Houran and Lange (2004) posit that this attributional process may serve the adaptive function of reducing fear. Another important source of variance might stem from exceptional experiences of the individual yielding convictions beyond what can be explained by rational thought (Fach et al., 2013).

This reasoning paves the way to consider the motivational and experiential structure behind paranormal beliefs in a systematic fashion. In social psychology, three general motivational principles are considered ubiquitous and of fundamental importance in driving human behavior: Striving for mastery, seeking connectedness, and valuing me and mine (e.g., Smith and Mackie, 2000). Based on results from a qualitative study, Betsch et al. (2020) found that paranormal beliefs were functional with regard to two fundamental motives, striving for mastery and valuing me and mine. Moreover, paranormal beliefs and their corresponding experiences help the individual to set behavioral goals and lead a meaningful life. However, on the negative side, participants frequently reported instances of exclusion and social stigmatization when revealing their beliefs to others. Further investigation of the dynamics of motivations and their effects on adaptation to the social world will help us to better understand why people endorse paranormal beliefs despite the proliferation of rationalism and scientific method in the modern age.

## 5 Conclusion

A skeptical person may immediately reject a statement if it violates a rule. Whereas open-minded, emotional people might be inspired to make sense of what seems odd at first glance. They may engage in associative, generic thought rather than in a bureaucratic, meticulous examination of the given information. Humans are capable of telling fictions. These fictions transcend the given and enhance experience with meaning, imagination, and emotion. In its essence, a good story widens our horizon. As such, story-telling is a virtue and not a deficit. Yet, if story seeking happens without reasoned review, the line between fiction and evidence-based knowledge becomes blurred.

## Declarations

### Author contribution statement

T. Betsch: Conceived and designed the experiments; Analyzed and interpreted the data; Wrote the paper.

L. Aßmann: Conceived and designed the experiments; Performed the experiments; Analyzed and interpreted the data.

A. Glöckner: Performed the experiments; Contributed reagents, materials, analysis tools or data.

### Funding statement

This research did not receive any specific grant from funding agencies in the public, commercial, or not-for-profit sectors.

### Competing interest statement

The authors declare no conflict of interest.

### Additional information

No additional information is available for this paper.

## Figures and Tables

**Table A:** Examples of bivariate correlations between paranormal beliefs (PB) and predictor variables.

Contructs	Effect Size	Authors
PB & Neuroticism	*r* = -.14, *p* < .05	Henningsgaard and Arnau (2008)
PB & Agreeableness	*r* = .24, *p* < .01 β = .20, *p* = .03 *r* = .25, *p* < .01	Schnell (2012)^1^ Henningsgaard and Arnau (2008)
PB & Extraversion	*r* = .21, *p* < .01 *r* = .17, *p* < .01	Schnell (2012)^1^ Henningsgaard and Arnau (2008)
PB & Conscientiousness	*r* = .26, *p* < .01 β = .18, *p* < .05 *r* = -.21, *p* < .05 β = -.19, *p* < .05 (Superstition subscale)	Henningsgaard and Arnau (2008) Williams and Roberts (2016)^8^
PB & Openness	*r* = .32, *p* < .01 β = .26, *p* = .003 *r* = -.29, *p* < .001 β = -.22, *p* < .05 (Superstition subscale) *r* = -.22, *p* < .01 (Precognition subscale)	Schnell (2012)^1^ Williams and Roberts (2016)^8^
PB & Honesty/Humility	*r* = -.19, *p* < .05 (Superstition subscale) *r* = -.23, *p* < .01 β = -.25, *p* < .01 (Precognition subscale)	Williams and Roberts (2016)^8^
PB & Education	η^2^ = .016–.034 (vocational school vs. university)	Aarnio and Lindeman (2005)^2^
PB & Cognitive Ability	*r* = .50, *p* < .01	Musch and Ehrenberg (2002)^3^
PB & Numeracy PB& Probabilistic Reasoning Errors	*r* = -.12 – -.46, *p* < .01 *r* = -.22, *p* < .01 no significant effect η^2^ = .22–.27, *p* < .001 *r* = .22, *p* < .05	Dagnall et al. (2014) Hergovich and Arendasy (2005) Brotherton and French (2014)^4^ Rogers et al. (2009) Musch and Ehrenberg (2002)^3^
PB & Ontological Confusion	*r* = .46, *p* < .001	Lindeman et al. (2015)^5^
PB& Need for Cognition	*r* = .35, *p* < .01	Gray and Gallo (2016)^6^
PB & Illusory Pattern Perception/Perception of Randomness	*r* = .38, *p* < .001 *r* = -.18 – -.43, *p* < .01 *r* = -.32, *p* < .01	van Prooijen et al. (2017) Dagnall et al. (2014) Dagnall et al. (2007)
PB & Cognitive Style	*r* = -.312, *p* < .01 *r* = -.14, *p* < .01 (analytical thinking) *r* = .34, *p* < .001 (intuitive thinking)	Gray and Gallo (2016)^6^ Aarnio and Lindeman (2005)^2^
PB & Death Anxiety	*r* = -.375, *p* < .001 No significant effect	Rasmussen and Johnson (1994) Henrie and Patrick (2014)^7^, Wink (2006)
PB & Life Satisfaction	*r* = .347, *p* < .01	Gray and Gallo (2016)^6^
PB & Gender	η^2^ = .028	Aarnio and Lindeman (2005)^2^

*Note.* Other analyzed factors: ^1^ horizontal self-transcendence, self-actualization order, well-being, and relatedness; ^2^ discipline, length of education, gender, analytical and intuitive thinking; ^3^ cognitive ability and probability misjudgement; ^4^ conspiracy theories, conjunction fallacy; ^5^ metalizing abilities (affective empathy, cognitive empathy, empathy abilities, hyper-empathizing), promiscuous teleology; ^6^ episodic memory accuracy and distortion, analytical thinking, absorption, dissociative experiences, need for cognition; ^7^ age, gender, religious belief, organizational religiousness, religious meaning, religious doubt.

Comments: ^3^ cognitive ability operationalized as “Abiturnote” (A-levels grade) – higher grade represents lower cognitive ability; ^8^ no correlation for the overall Revised Paranormal Belief Scale.

**Table B:** Correlation of the personality predictor variables and the criterion.

	Paranormal Beliefs	HH	Em	eX	Ag	Con	Op
HH	-.009						
Em	.150^∗∗^	.063					
eX	.043	.057	-.185^∗∗^				
Ag	.042	.274^∗∗^	-.184^∗∗^	.233^∗∗^			
Con	-.144^∗∗^	.197^∗∗^	.001	.198^∗∗^	.009		
Op	.074	.228^∗∗^	-.022	.195^∗∗^	.084^∗^	.107^∗∗^	
Altr.	.087^∗^	.546^∗∗^	.364^∗∗^	.230^∗∗^	.399^∗∗^	.189^∗∗^	.250^∗∗^

*Note.* N = 599 ∗∗The correlation is significant at the .01 level (2-tailed). ∗The correlation is significant at the .05 level (2-tailed).

**Table C:** Correlation of the ability predictor variables and the criterion.

	Paranormal Beliefs	Education	Ontological Confusion	Causality Understanding
Education	-.082^∗^			
Ontological Confusion	.328^∗∗^	-.130^∗∗^		
Causality Understanding	-.173^∗∗^	.127^∗∗^	-.169^∗∗^	
Intelligence	-.220^∗∗^	.246^∗∗^	-.159^∗∗^	.119^∗∗^

*Note.* N = 599 ∗∗The correlation is significant at the .01 level (2-tailed). ∗The correlation is significant at the .05 level (2-tailed).

**Table D:** Correlation of the person predictor variables and the criterion.

	Paranormal Beliefs	Lifesatisfaction	IPP	Death Anxiety	Age
Lifesatisfaction	.009				
Illusory Pattern Perception	.128^∗∗^	-.018			
Death Anxiety	.145^∗∗^	-.193^∗∗^	.063		
Age	-.026	.041	-.100^∗^	-.074	
Sex	.247^∗∗^	.022	.022	.160^∗∗^	-.086^∗^

*Note.* N = 599 ∗∗The correlation is significant at the .01 level (2-tailed). ∗The correlation is significant at the .05 level (2-tailed).

**Table E:** Descriptives of the predictor variables and the criterion.

	Min.	Max.	Mean	SD
Honesty-Humility	1.06	5	3.58	.72
Emotionality	1.13	4.69	3.23	.59
eXtraversion	1.38	4.88	3.33	.66
Agreeableness	1.06	4.56	3.01	.59
Conscientiousness	1.31	4.81	3.57	.57
Openness to Experience	1.75	4.88	3.48	.60
Ontological Confusion	1	4.07	2.47	.59
Causality Understanding	1	6	5.35	1.10
Numeracy	1	15	12.26	1.95
Cognitive Style	0	3	1.75	1.13
Ambiguity Tolerance	2.23	6.23	4.49	.75
Need for Cognitive Closure	1.25	5.56	3.24	.70
Need for Cognition	1.75	7	5.19	.92
Epistemological Prudence	2.67	7	4.91	.67
Illusory Pattern Perception	1	5.60	2.42	1.32
Lifesatisfaction	1	11	7.73	2.35
Death Anxiety	0	15	8.04	2.86
Paranormal beliefs	1	6	2.01	.99

*Note.* N = 599.

## Appendix A Supplementary data

Predictors of Paranormal Beliefs Supplement_English Predictors of Paranormal Beliefs Supplement_English

## Footnotes

1 There are a number of additional factors that foster paranormal beliefs, e.g., experiences (Fach et al., 2013), attribution (Irwin et al., 2013; Lange et al., 2019), functional aspects such as fear reduction (Lange and Houran, 1999), increasing mastery of the situation, and enhancing self-evaluation (Betsch et al., 2020). These factors, however, are beyond the focus of this study.

2 The study was preregistered on OSF: DOI: 10.17605/OSF.IO/RGJ3E. Data, material, and syntax can be found there and in the online supplement. The study also assessed other criterion variables (e.g., religious beliefs). Results for those variables are not reported in this paper. Political orientation was measured as a potential predictor. Due to a very high number of missing data, political orientation was not included in data analyses.

3 The pool consisted of a total of N = 1,632 participants (62% students, 39% of whom are psychology majors; 49% employed), who were recruited for participation in multiple payed studies via University of Hagen email lists and advertisements.

4 Ontological confusions are defined as category mistakes in which properties of living and lifeless entities are mixed (cf., e.g., Lindeman et al., 2015).

5 The correlation between female and IQ in the overall database was similar (*r* = -.19), which might be due to the specific sample.

6 Accordingly, religious propositions and dogma do not represent an exception. Accordingly, someone who lacks ability and motivation for rational analysis may also be susceptible to accept religious beliefs, as he or she does accept paranormal beliefs (cf. e.g., Lindeman et al., 2015). However, a detailed discussion of this notion is beyond the scope of this paper.
