# Supplementary material for: Paranormal beliefs and individual differences: story seeking without reasoned review
Source: Heliyon. 2020 Jun 30;6(6):e04259. doi: 10.1016/j.heliyon.2020.e04259 (PMC7330497; doi:10.1016/j.heliyon.2020.e04259)
Supplement: Predictors of Paranormal Beliefs Supplement_English [file mmc1.docx]

Content

[HEXACO 2](#_Toc43108921)

[Ontological Confusion 6](#_Toc43108922)

[Numeracy 7](#_Toc43108923)

[Causality Understanding 9](#_Toc43108924)

[Ambiguity Tolerance 10](#_Toc43108925)

[Need for Cognitive Closure 11](#_Toc43108926)

[Epistemological Prudence 12](#_Toc43108927)

[Need for Cognition 13](#_Toc43108928)

[Life Satisfaction 14](#_Toc43108929)

[Illusory Pattern Perception 15](#_Toc43108930)

[Death Anxiety 16](#_Toc43108931)

[Cognitive Style 17](#_Toc43108932)

[Paranormal Beliefs 18](#_Toc43108933)

[Overview 19](#_Toc43108934)

[References 21](#_Toc43108935)

# HEXACO

| **Scale Name** | **HEXACO-PI-R** |
| --- | --- |
| Source | Lee & Ashton (2018) |
| Language | English |
| Original Language | English |
| Number of Items | 100 |
| Answer format | Rating-Scale |
| Weidth of Scale | - 1. (1 = strongly disagree, 2 = disagree, 3 = neutral, 4 = agree, 5 = strongly agree) |

1. I would be quite bored by a visit to an art gallery.
2. I clean my office or home quite frequently.
3. I rarely hold a grudge, even against people who have badly wronged me.
4. I feel reasonably satisfied with myself overall
5. I would feel afraid if I had to travel in bad weather conditions.
6. If I want something from a person I dislike, I will act very nicely toward that person in order to get it.
7. I'm interested in learning about the history and politics of other countries.
8. When working, I often set ambitious goals for myself.
9. People sometimes tell me that I am too critical of others.
10. I rarely express my opinions in group meetings.
11. I sometimes can't help worrying about little things.
12. If I knew that I could never get caught, I would be willing to steal a million dollars.
13. I would like a job that requires following a routine rather than being creative.
14. I often check my work over repeatedly to find any mistakes.
15. People sometimes tell me that I'm too stubborn.
16. I avoid making "small talk" with people.
17. When I suffer from a painful experience, I need someone to make me feel comfortable.
18. Having a lot of money is not especially important to me.
19. I think that paying attention to radical ideas is a waste of time.
20. I make decisions based on the feeling of the moment rather than on careful thought.
21. People think of me as someone who has a quick temper.
22. I am energetic nearly all the time.
23. I feel like crying when I see other people crying.
24. I am an ordinary person who is no better than others.
25. I wouldn't spend my time reading a book of poetry.
26. I plan ahead and organize things, to avoid scrambling at the last minute.
27. My attitude toward people who have treated me badly is "forgive and forget".
28. I think that most people like some aspects of my personality.
29. I don’t mind doing jobs that involve dangerous work.
30. I wouldn't use flattery to get a raise or promotion at work, even if I thought it would succeed.
31. I enjoy looking at maps of different places.
32. I often push myself very hard when trying to achieve a goal.
33. I generally accept people’s faults without complaining about them.
34. In social situations, I'm usually the one who makes the first move.
35. I worry a lot less than most people do.
36. I would be tempted to buy stolen property if I were financially tight.
37. I would enjoy creating a work of art, such as a novel, a song, or a painting.
38. When working on something, I don't pay much attention to small details.
39. I am usually quite flexible in my opinions when people disagree with me.
40. I enjoy having lots of people around to talk with.
41. I can handle difficult situations without needing emotional support from anyone else.
42. I would like to live in a very expensive, high-class neighborhood.
43. I like people who have unconventional views.
44. I make a lot of mistakes because I don't think before I act.
45. I rarely feel anger, even when people treat me quite badly.
46. On most days, I feel cheerful and optimistic.
47. When someone I know well is unhappy, I can almost feel that person's pain myself.
48. I wouldn’t want people to treat me as though I were superior to them.
49. If I had the opportunity, I would like to attend a classical music concert.
50. People often joke with me about the messiness of my room or desk.
51. If someone has cheated me once, I will always feel suspicious of that person.
52. I feel that I am an unpopular person.
53. When it comes to physical danger, I am very fearful.
54. If I want something from someone, I will laugh at that person's worst jokes.
55. I would be very bored by a book about the history of science and technology.
56. Often when I set a goal, I end up quitting without having reached it.
57. I tend to be lenient in judging other people.
58. When I'm in a group of people, I'm often the one who speaks on behalf of the group.
59. I rarely, if ever, have trouble sleeping due to stress or anxiety.
60. I would never accept a bribe, even if it were very large.
61. People have often told me that I have a good imagination.
62. I always try to be accurate in my work, even at the expense of time.
63. When people tell me that I’m wrong, my first reaction is to argue with them.
64. I prefer jobs that involve active social interaction to those that involve working alone.
65. Whenever I feel worried about something, I want to share my concern with another person.
66. I would like to be seen driving around in a very expensive car.
67. I think of myself as a somewhat eccentric person.
68. I don’t allow my impulses to govern my behavior.
69. Most people tend to get angry more quickly than I do.
70. People often tell me that I should try to cheer up.
71. I feel strong emotions when someone close to me is going away for a long time.
72. I think that I am entitled to more respect than the average person is.
73. Sometimes I like to just watch the wind as it blows through the trees.
74. When working, I sometimes have difficulties due to being disorganized.
75. I find it hard to fully forgive someone who has done something mean to me.
76. I sometimes feel that I am a worthless person.
77. Even in an emergency I wouldn't feel like panicking.
78. I wouldn't pretend to like someone just to get that person to do favors for me.
79. I’ve never really enjoyed looking through an encyclopedia.
80. I do only the minimum amount of work needed to get by.
81. Even when people make a lot of mistakes, I rarely say anything negative.
82. I tend to feel quite self-conscious when speaking in front of a group of people.
83. I get very anxious when waiting to hear about an important decision.
84. I’d be tempted to use counterfeit money, if I were sure I could get away with it.
85. I don't think of myself as the artistic or creative type.
86. People often call me a perfectionist.
87. I find it hard to compromise with people when I really think I’m right.
88. The first thing that I always do in a new place is to make friends.
89. I rarely discuss my problems with other people.
90. I would get a lot of pleasure from owning expensive luxury goods.
91. I find it boring to discuss philosophy.
92. I prefer to do whatever comes to mind, rather than stick to a plan.
93. I find it hard to keep my temper when people insult me.
94. Most people are more upbeat and dynamic than I generally am.
95. I remain unemotional even in situations where most people get very sentimental.
96. I want people to know that I am an important person of high status.
97. I have sympathy for people who are less fortunate than I am.
98. I try to give generously to those in need.
99. It wouldn’t bother me to harm someone I didn’t like.
100. People see me as a hard-hearted person.

# Ontological Confusion

| **Scale Name** | **Core Knowledge Confusions Scale** |
| --- | --- |
| Source | Lindeman et al. (2015)  **NOTE.** Scale has not been validated. The following items were used in Lindeman, et al. (2015) because the data used in the earlier articles show that they are most predictive of paranormal (supernatural, magical, superstitious) beliefs. |
| Language | English |
| Original Language | English |
| Number of Items | 20 (14 Scale Items, 6 Filler Items [marked in bold]) |
| Answer format | Rating-Scale |
| Weidth of Scale | 1 (completely metaphorical) – 5 (completely literal) |

1. Rock lives long
2. Force lives in the universe
3. Force aims to influence
4. Earth wants water
5. Planets know things
6. The sky hears the thunder.
7. Flowers want light
8. Plants know the seasons
9. Home knows its residents
10. Furniture wants a home
11. House knows its history
12. Mind touches the other
13. A plan lives in the nature
14. Fear poisons man.
15. **Good memory is a mine**
16. **Howling wind is a flute**
17. **Distressed person is a prisoner**
18. **Rainless weather is dry**
19. **Bad drawing is a scribble**
20. **Flowing water is liquid**

# Numeracy

| **Scale Name** | **Numeracy** |
| --- | --- |
| Source | Peters et al. (2007) |
| Language | English |
| Original Language | English |
| Number of Items | 14 |
| Answer format | - |
| Weidth of Scale | - |

1. Imagine that we roll a fair, six-sided die 1,000 times. Out of 1,000 rolls, how many times do you think the die would come up even?
2. In the Big Bucks Lottery, the chances of winning a $10 prize are 1%. What is your best guess about how many people would win a $10 prize if 1,000 people each buy a single ticket from Big Bucks?
3. In the Acme Publishing Sweepstakes, the chance of winning a car is 1 in 1,000. What percentage of tickets of Acme Publishing Sweepstakes wins a car?
4. Which of the following numbers represents the biggest risk of getting a disease?

□ 1 in 100 □ 1 in 1000 □ 1 in 10

1. Which of the following numbers represents the biggest risk of getting a disease?

□ 1% □ 10% □ 5%

1. If Person A’s risk of getting a disease is 1% in 10 years, and Person B’s risk is double that of A’s, what is B’s risk?
2. If Person A’s chance of getting a disease is 1 in 100 in 10 years, and person B’s risk is double that of A, what is B’s risk?
3. If the chance of getting a disease is 10%, how many people would be expected to get the disease

□ out of 100? ___

□ out of 1000? ___

1. If the chance of getting a disease is 20 out of 100, this would be the same as having a ____% chance of getting the disease.
2. The chance of getting a viral infection is .0005. Out of 10,000 people, about how many of them are expected to get infected?
3. Which of the following numbers represents the biggest risk of getting a disease?

□ 1 von 12 □ 1 von 37

1. Suppose you have a close friend who has a lump in her breast and must have a mammogram. Of 100 women like her, 10 of them actually have a malignant tumor and 90 of them do not. Of the 10 women who actually have a tumor, the mammogram indicates correctly that 9 of them have a tumor and indicates incorrectly that 1 of them does not. Of the 90 women who do not have a tumor, the mammogram indicates correctly that 81 of them do not have a tumor and indicates incorrectly that 9 of them do have a tumor. The table below summarizes all of this information. Imagine that your friend tests positive (as if she had a tumor), what is the likelihood that she actually has a tumor?
2. Imagine that you are taking a class and your chances of being asked a question in class are 1% during the first week of class and double each week thereafter (i.e., you would have a 2% chance in Week 2, a 4% chance in Week 3, an 8% chance in Week 4). What is the probability that you will be asked a question in class during Week 7?
3. Suppose that 1 out of every 10,000 doctors in a certain region is infected with the SARS virus; in the same region, 20 out of every 100 people in a particular at-risk population also are infected with the virus. A test for the virus gives a positive result in 99% of those who are infected and in 1% of those who are not infected. A randomly selected doctor and a randomly selected person in the at-risk population in this region both test positive for the disease. Who is more likely to actually have the disease?

□ both

□ doctor

□ at-risk person

# Causality Understanding

| **Scale Name** | **Tom in South America** |
| --- | --- |
| Source | Betsch, Wünsche & Klodt (2017)^a^ |
| Language | English |
| Original Language | German |
| Number of Items | 6 |
| Answer format | Yes / No |
| Weidth of Scale | - |

Tom is on vacation in South America. The weather is marvellous, the landscape breath-taking. If it were not for those little bugs that, in the evenings, would come onto the porch in crowds. They dart for the dinner and deliver nasty pricks when they land on the skin.

The gardener, an old Indigo who works at the facility, has an advice. He gives Tom a kind of incense sticks, which consist of a mix of herbs. He says he has distributed them to the other guests at the facility who are struggling with the bugs, as well. On his last evening, Tom gives the sticks a try. And indeed – not a single bug flies onto the porch.

What do you need to know to assess whether the incense sticks were the actual reason for the absence of bugs?

Please indicate for every statement, if you agree [Yes] or disagree [No].

1. I need to know whether the bugs also stayed away from other guests who did not use the incense sticks that evening.
2. I need to know what other guests have to say about the gardener’s credibility. (reverse)
3. Tom’s account is enough. After all, he witnessed the effect of the sticks himself. (reverse)
4. I need to know whether the bugs also stayed away from other guests who, too, used the incense sticks that evening.
5. I need to know whether the bugs stay away on other days when using the incense sticks and whether they come back when the sticks are not used.
6. I do not need any more information. I trust the indigenous people and their knowledge. (reverse)

# Ambiguity Tolerance

| **Scale Name** | **Multiple Stimulus Types Ambiguity Tolerance Scale II** |
| --- | --- |
| Source | McLain (2009) |
| Language | English |
| Original Language | English |
| Number of Items | 13 |
| Answer format | Rating-Scale |
| Weidth of Scale | 1 – 7 |

1. I do not tolerate ambiguous situations well. (reverse)
2. I would rather avoid solving a problem that must be viewed from several different perspectives. (reverse)
3. I try to avoid situations that are ambiguous. (reverse)
4. I prefer familiar situations to new ones. (reverse)
5. Problems that cannot be considered from just one point of view are a little threatening. (reverse)
6. I avoid situations that are too complicated for me to easily understand. (reverse)
7. I am tolerant of ambiguous situations.
8. I enjoy tackling problems that are complex enough to be ambiguous.
9. I try to avoid problems that do not seem to have only one “best” solution.
10. I generally prefer novelty over familiarity.
11. I dislike ambiguous situations. (reverse)
12. I find it hard to make a choice when the outcome is uncertain. (reverse)
13. I prefer a situation in which there is some ambiguity.

# Need for Cognitive Closure

| **Scale Name** | **Kurzskala zur Erfassung des Bedürfnisses nach kognitiver Geschlossenheit** |
| --- | --- |
| Source | Schlink & Walther (2007), adaption of Webster & Kruglanski (1994) |
| Language | English |
| Original Language | German |
| Number of Items | 16 |
| Answer format | Rating-Scale |
| Weidth of Scale | 1 (totally disagree) – 6 (totally agree) |

1. I do not like when a person’s statement is ambiguous.
2. In my opinion, it is a waste of time to consider other possible solutions after finding a solution to a problem.
3. I do not like unpredictable situations.
4. I find is exciting not to know what life will bring you.
5. Looking at a problem from different angles only leads to confusion.
6. Generally, I am not looking for alternative solutions to problems for which I already have a solution.
7. I prefer the company of good friends because I know what to expect from them.
8. I feel uncomfortable if I fail to give a quick response to problems I am facing.
9. I prefer activities where it is always clear what needs to be done and how it has to be done.
10. Generally, when I need to solve a problem, I do not waste time considering the different points of view.
11. I like tasks where it is still unclear how the exact solution looks like.
12. I love the uncertainty and surprise that is often in everyday life.
13. Any solution to a problem is better than staying in a state of uncertainty.
14. I prefer things that I am used to over things that I do not know and that I cannot predict.
15. In general, I avoid taking part in discussions on ambiguous and controversial topics.
16. I prefer to choose the first possible solution instead of thinking about what decision I should make.

# Epistemological Prudence

| **Scale Name** | **Epistemological Prudence Scale** |
| --- | --- |
| Source | Betsch (2017) |
| Language | English |
| Original Language | German |
| Number of Items | 12 |
| Answer format | Rating-Scale |
| Weidth of Scale | 1 (totally disagree) – 7 (totally agree) |

1. There are final, irrefutable truths. (reverse)
2. All our knowledge is provisional.
3. We achieve progress of knowledge by looking for evidence to support our assumptions.
4. Insights and convictions have different likelihoods of being valid.
5. We achieve progress of knowledge by examining the conditions under which our assumptions do not hold true.
6. After assuming a conviction or insight, you should not be deterred by refutation. (reverse)
7. Truth must be defended from objection. (reverse)
8. You should always strive for critical examination of insights and convictions.
9. No insight or conviction may ever claim to be absolutely valid. (reverse)
10. Common sense allows anyone to judge the validity of insights and convictions by themselves. (reverse)
11. You should always be aware of what you don’t know.
12. The validity of insights and convictions can only be assessed when everything that has led to their discovery is disclosed.

# Need for Cognition

| **Scale Name** | **Need for Cognition: Skala zur Erfassung von Engagement und Freude bei Denkaufgaben** |
| --- | --- |
| Source | Bless et al. (1994), adaption of Cacioppo & Petty (1982) |
| Language | English |
| Original Language | German |
| Number of Items | 16 |
| Answer format | Rating-Scale |
| Weidth of Scale | 1 (trifft überhaupt nicht zu) – 7 (trifft ganz genau zu) |

1. I really enjoy a task that involves coming up with new solutions to problems.
2. I would prefer a task that is intellectual, difficult, and important to one that is somewhat important but does not require much thought.
3. I tend to set goals that can be accomplished only by expending considerable mental effort.
4. The idea of relying on thought to make my way to the top does not appeal to me.
5. I find it especially satisfying to complete an important task that required a lot of thinking and mental effort.
6. I prefer to think about small, daily projects to long-term ones.
7. I would rather do something that requires little thought than something that is sure to challenge my thinking abilities.
8. I find little satisfaction in deliberating hard and for long hours.
9. I think primarily because I have to.
10. I do not like to have the responsibility of handling a situation that requires a lot of thinking.
11. Thinking is not my idea of fun.
12. I try to anticipate and avoid situations where there is a likely chance I will have to think in depth about something.
13. I prefer my life to be filled with puzzles that I must solve.
14. I would prefer complex to simple problems.
15. Simply knowing the answer rather than understanding the reasons for the answer to a problem is.
16. It is enough for me that something gets the job done, I don't care how or why it works.

# Life Satisfaction

| **Scale Name** | **Short scale Life satisfaction-1 [Kurzskala Lebenszufriedenheit-1 (L-1)]** |
| --- | --- |
| Source | Beierlein et al. (2015) |
| Language | English |
| Original Language | German |
| Number of Items | 1 |
| Answer format | Rating-Scale |
| Weidth of Scale | 0 (not at all satisfied) – 10 (totally satisfied) |

How satisfied are you currently with your life, all things considered?

(Original: Wie zufrieden sind Sie gegenwärtig, alles in allem, mit ihrem Leben?)

# Illusory Pattern Perception

| **Scale Name** | **Illusory Pattern Perception** |
| --- | --- |
| Source | Van Prooijen et al. (2017) |
| Language | English |
| Original Language | English |
| Number of Items | 11 |
| Answer format | Rating-Scale |
| Weidth of Scale | 1 (completely random) – 7 (completely determined) |

**How random are these coin flips?**

Below you each time see the results of the same coin being flipped 10 times ("H" means Heads, and "T" means Tails). Please rate the extent to which you see a pattern. If you believe the coin flip results are completely random, answer "1". If you believe the coin flip results are completely determined (for instance because of a biased coin, or if the results were rigged), please answer "7".

1. H T H H H H H H T H
2. H T H H T T T T H H
3. H H H T T T T T H H
4. H T H H H T H T H H
5. H T T H H T T T T T
6. H T T H T H H H T T
7. T H H T T T H H T H
8. H T H H H T H T H T
9. T T H T T H T H H T
10. T T H T T T T H T T
11. Now, imagine that the above items represent 100 consecutive throws with the same coin. Please again rate how random or determined the outcomes are.

# Death Anxiety

| **Scale Name** | **Death Anxiety Scale** |
| --- | --- |
| Source | Templer (1970) |
| Language | English |
| Original Language | English |
| Number of Items | 15 |
| Answer format | Yes / No |
| Weidth of Scale | - |

1. I am very much afraid to die.

2. The thought of death seldom enters my mind. (reverse)

3. It doesn’t make me nervous when people talk about death. (reverse)

4. I dread to think about having to have an operation.

5. I am not at all afraid to die. (reverse)

6. I am not particularly afraid of getting cancer. (reverse)

7. The thought of death never bothers me. (reverse)

8. I am often distressed by the way time flies so very rapidly.

9. I fear dying a painful death.

10. The subject of life after death troubles me greatly.

11. I am really scared of having a heart attack.

12. I often think about how short life really is.

13. I shudder when I hear people talking about a World War III.

14. The sight of a dead body is horrifying to me.

15. I feel that the future holds nothing for me to fear. (reverse)

# Cognitive Style

| **Scale Name** | **Cognitive Reflection Test (CRT)** |
| --- | --- |
| Source | Frederick (2005) |
| Language | English |
| Original Language | English |
| Number of Items | 3 |
| Answer format | - |
| Weidth of Scale | - |

1. A bat and a ball cost $1.10 in total. The bat costs a dollar more than the ball. How much does the ball cost? ____ cents [Correct answer 5 cents; intuitive answer 10 cents]
2. If it takes 5 machines 5 minutes to make 5 widgets, how long would it take 100 machines to make 100 widgets? ____ minutes [Correct answer ¼ 5 minutes; intuitive answer ¼ 100 minutes]
3. In a lake, there is a patch of lily pads. Every day, the patch doubles in size. If it takes 48 days for the patch to cover the entire lake, how long would it take for the patch to cover half of the lake? ____ days [Correct answer ¼ 47 days; intuitive answer ¼ 24 days] The score on these original three items will be designated CRT3 in our study.

# Paranormal Beliefs

| **Scale name** | **Paranormal beliefs** |
| --- | --- |
| Source | Betsch, Wünsche & Klodt (2017)^b^ |
| Original language | German |
| Number of items | 5 |
| Answer format | Rating-Scale |
| Scale range | 1 (not at all) – 6 (very much) |

Please state how much you believe in the following:

1. Esoterism (e.g. chakra, feng shui, reiki, energy healing…)
2. Spirituality (e.g. channelling, reincarnation, past life regression…)
3. Supernatural beings (e.g. ghosts, light beings, spirits, demons ...)
4. Astrology (e.g. horoscope, tarot cards...)
5. Magic (e.g. sorcery, witchcraft, voodoo...)

# Overview

Table I

*Predictor variables*

| Predictors | Measures & Example Items |
| --- | --- |
| Big Six | HEXACO-PI-R (Lee & Ashton, 2016) |
| - Honesty/ Humility | “If I knew that I could never get caught, I would be willing to steal a million dollars.” |
| - Emotionality | “I sometimes can’t help worrying about little things.” |
| - Extraversion | “In social situations, I’m usually the one who makes the first move.” |
| - Agreeableness | “Most people tend to get angry more quickly than I do.” |
| - Conscientiousness | “I often push myself very hard when trying to achieve a goal.” |
| - Openness to New Experiences | “I have a good imagination.” |
| - Altruism | “I try to give generously to those in need.” |
| Cognitive Ability (IQ) | 10-Minute-IQ-Test (Musch et al., 2009)  “A boy is 7 years old and his sister is twice as old as him. How old will his sister be when the boy is 11 years old?” |
| Education | *Self report* |
| Ontological Confusion | Core Knowledge Confusions Scale (Lindeman & Aarnio, 2007; Lindeman et al., 2015)  “Mind touches another” “House knows its history” |
| Numeracy | DR-Numeracy Test (Peters, Dieckmann, Dixon, Hibbard, & Mertz, 2007)  “Which of the following numbers represents the biggest risk of getting a disease? 1%, 10%, 5%” |
| Causality Understanding | Scenario (Wünsche, 2017; Klodt, 2017)  *The scenario describes an incidence of a seemingly causal relationship between a repellant and the absence of bugs. Participants were asked whether the coincidence of the treatment (repellant) and the effect (absence of bugs) suffices as proof of a causal relationship. Moreover, they were presented with methods for testing causality, which resemble an experimental design.* |
| Ambiguity Tolerance | MSTAT-II (McLain, 2009)  “I prefer familiar situations to new ones.” |
| Need for Cognitive Closure | Short scale for the assessment of need for cognitive closure [Kurzskala zur Erfassung des Bedürfnisses nach kognitiver Geschlossenheit] (Schlink & Walther, 2007)  “In general, I don‘t look for alternative solutions if I already have a solution for the problem.” |
| Epistemological Prudence | Epistemological Prudence Scale (Betsch, 2017)  “We obtain new knowledge by examining the conditions under which our assumptions do not hold true.” |
| Need for Cognition | Scale for the assessment of need for cognition [Skala zur Erfassung von Engagement und Freude bei Denkaufgaben] (Bless et al., 1994)  “I think it is satisfying to finish an important task which needed much thinking and cognitive effort.” |
| Life Satisfaction | Short scale life satisfaction-1 [Kurzskala Lebenszufriedenheit-1] (Beierlein et al., 2014)  “All in all, how satisfied are you with your life?” |
| Illusory Pattern Perception | 10 random coin tosses (Van Prooijen et al., 2017)  “How random are these coin tosses? 1. K Z K K K K K K Z K” |
| Death Anxiety | Death Anxiety Scale (Templer, 1970)  “I am very much afraid to die.” |
| Cognitive Style | Cognitive reflection test (Frederick, 2005)  “A bat and a ball cost 1.10 €. The bat is 1.00€ more expensive than the ball. How much does the bat cost?” |
| Age | *Self report* |
| Gender | *Self report* |

# References

Beierlein, C., Kovaleva, A., László, Z., Kemper, C. J., Rammstedt, B. (2015). Kurzskala zur Erfassung der Allgemeinen Lebenszufriedenheit (L-1). Zusammenstellung sozialwissenschaftlicher Items und Skalen. <https://doi.org/10.6102/zis229.>

Betsch, T. (2017). *Epistemological Prudence Scale*. University of Erfurt: Unpublished manuscript.

Betsch, T., Wünsche, K. & Klodt, S. (2017)^a^. *An Assessment of* *Causality Understandig*. University of Erfurt. Unpublished manuscript.

Betsch, T., Wünsche, K. & Klodt, S. (2017)^b^. *Irrational Belief Scale*. University of Erfurt. Unpublished manuscript.

Bless, H., Wänke, M., Bohner, G., Fellhauer, R. F., & Schwarz, N. (1994). Need for cognition: eine Skala zur Erfassung von Engagement und Freude bei Denkaufgaben; [Need for cognition: a scale measuring engagement and happiness in cognitive tasks]. *Zeitschrift für Sozialpsychologie*, *25*, 147-154.

Frederick, S. (2005). Cognitive reflection and decision making. *Journal of Economic Perspectives*, *19*, 25–42. <https://doi.org/10.1257/089533005775196732>

Lee, K., & Ashton, M. C. (2018). Psychometric properties of the HEXACO-100. *Assessment, 25*, 543-556.

McLain, D. L. (2009). Evidence of the properties of an ambiguity tolerance measure: The Multiple Stimulus Types Ambiguity Tolerance Scale-II (MSTAT-II). *Psychological Reports*, *105*, 975–988. <https://doi.org/10.2466/pr0.105.3.975-988>

Musch, J., Ostapczuk, M., Hilbig, B. E., Auer, T.S., Brandt, M., Cüpper, L., Erdfelder, E. & Undorf, M. (2009). 10-Minuten-Test. Unpublished Test, University of Düsseldorf.

Peters, E., Dieckmann, N., Dixon, A., Hibbard, J. H., & Mertz, C. K. (2007). Less is more in presenting quality information to consumers. *Medical Care Research and Review*, *64*, 169-190.

Schlink, S., & Walther, E. (2007). Kurz und gut: Eine deutsche Kurzskala zur Erfassung des Bedürfnisses nach kognitiver Geschlossenheit. *Zeitschrift für Sozialpsychologie*, *38*, 153–161.

Templer, D. I. (1970). The construction and validation of a Death Anxiety Scale. *Journal of General Psychology, 82,* 165-77*.* <https://doi.org/10.1002/10.1080/00221309.1970.9920634>

van Prooijen, J.-W., Douglas, K. M., & De Inocencio, C. (2017). Connecting the dots: Illusory pattern perception predicts belief in conspiracies and the supernatural. *European Journal of Social Psychology, 48,* 320-335. <https://doi.org/10.1002/ejsp.2331>
